# Effect of combination fluoxetine and exercise on prefrontal BDNF, anxiety-like behavior and fear extinction in a female rat model of post-traumatic stress disorder (PTSD): a comparison with male animals

**DOI:** 10.1186/s12993-023-00204-z

**Published:** 2023-01-16

**Authors:** Sakineh Shafia, Farkhonde Nikkhah, Kobra Akhoundzadeh

**Affiliations:** 1 Immunogenetics Research Center, Department of Physiology, Mazandaran University of Medical Sciences, Sari, Iran; 2 Student Research Committee, Faculty of Medicine, Mazandaran University of Medical Sciences, Sari, Iran; 3 Faculty of Nursing and Midwifery, Qom University of Medical Sciences, Qom, Iran

**Keywords:** PTSD, Single prolonged stress, Exercise, Fluoxetine, sex

## Abstract

Despite significant differences between men and women in the symptoms of PTSD and the response to therapeutic interventions, most PTSD studies have been done on male subjects. Continuing our previous study in male rats, this study aimed at better understanding the effect of a combination therapy of exercise with fluoxetine on female PTSD rats. The results were then compared with our past findings in male animals. Female adult Wistar rats subjected to PTSD were treated with moderate treadmill exercise or fluoxetine, or a combination of both. PTSD was induced by the single prolonged stress (SPS) model. Elevated plus-maze (EPM), serum and prefrontal BDNF, and fear extinctions were evaluated. The results showed that exercise plus fluoxetine decreased anxiety-like behavior, improved fear extinction, and increased BDNF changes in female rats. The effects of exercise alone were comparable with those of combination therapy except that combination therapy was more effective on OAT (open arm entry). The majority of results in female rats, except for those of prefrontal BDNF, 4th extinction, and OAT, were similar to those of male rats as shown in our previous study. According to our findings, exercise as a safe and cost-effective intervention can be considered as a complementary efficient option for PTSD treatment in both sexes. To achieve better treatment outcomes in PTSD patient, considering sex differences is recommended.

## Introduction

Women are more susceptible to the experience of PTSD after a traumatic event compared to men [1]. Indeed, they are twice as likely as men to be affected by PTSD [2]. Also, there are significant differences between men and women in terms of PTSD symptoms and the effects of treatments [3–6]. In spite of sex differences in PTSD, our current knowledge of PTSD is mostly based on research on males, and not enough attention has been given to assess the effect of sex on traumatic stress [7].

Regardless of the several therapeutic approaches for PTSD, it is still a disorder without satisfying interventions for all cases of patients [8]. For example, it has been shown that fluoxetine as a known treatment for PTSD is not effective in combat veterans with severe, chronic PTSD [9]. Moreover, studies revealed that responses to treatment with fluoxetine may be different regarding sex [9, 10].

Anxiety and depression-like behaviors are core symptoms of PTSD [11] and exercise has been shown to have an anti-depressant-like property [12]. Exercise has been of interest in the treatment of depressive disorders because of its synergistic effect with antidepressants as well as being a relatively safe and low-cost intervention [12]. Based on the anti-depressant-like properties of exercise [12] it seems that the combination of exercise with fluoxetine may lead to greater therapeutic effects on PTSD recovery. It has been shown that a combination of fluoxetine and exercise, but not fluoxetine alone, increases neurogenesis in postpartum depression [13]. However, some studies did not confirm whether the combination of exercise and anti-depressant would result in an augmentative effect or not [12]. Moreover, it seems that the effects of exercise are related to the variables such as exercise intensity [6, 14] and the sex of subjects [15].

It is asserted that a full understanding of PTSD needs sex differences to be investigated in the responses to traumatic stress [7]. Since the effects of both fluoxetine and exercise on behavior are influenced by sex, the present study, following our previous research in male rats, was designed to assess the effect of the combination of exercise and fluoxetine on anxiety-like behavior and cellular alteration in female PTSD and to compare present results with our past findings in male animals.

## Methods and materials

### Animals

**Fig. 1:**
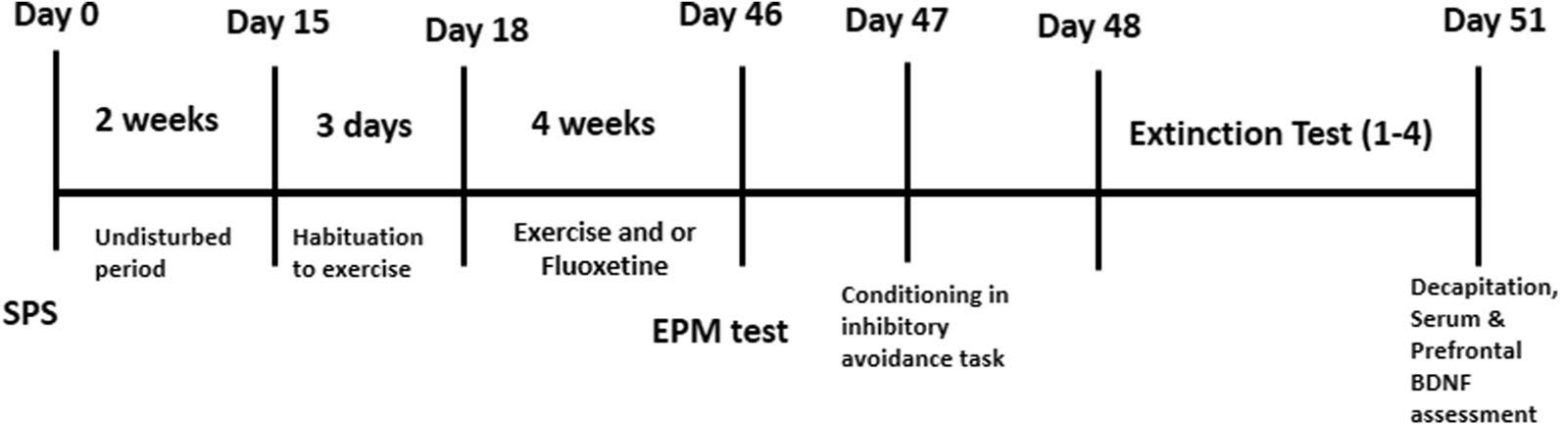
Timelines of experiments

Female adult Wistar rats (200–250 g BW) were bred, supplied, and housed at the animal house of Mazandaran University of Medical Science, Sari, Iran. Animals were kept in a temperature-controlled room (18–24 °C), with a 12/12 h light–dark cycle, and given ad libitum access to water and food. All experiments were performed in accordance with the Research Ethics Committee of Mazandaran University of Medical Science (ethical code numbers: IR.MAZUMS.REC.1399.7873, IR.MAZUMS.4.REC.1400.10267) and the Iran National Committee for Ethics in Biomedical Research. The animals were randomly allocated into 8 groups (each 7–10 rats); sham without receiving any intervention (NSPS/SED-VEH), sham received fluoxetine (NSPS/SED-FLX), sham did exercise (NSPS/EXC-VEH), sham received fluoxetine and did exercise (NSPS/ EXC-FLX), SPS without receiving any intervention (SPS/SED-VEH), SPS received fluoxetine (SPS/SED-FLX), SPS did exercise (SPS/EXC-VEH), SPS received fluoxetine and did exercise (SPS/EXC-FLX). The timeline of research is shown in Fig. 1.

### Single prolonged stress (SPS) induction

As illustrated in our previous study, SPS as a model of PTSD was induced in rats by being faced with three stressors in three phases [16]. Briefly, the rat in the first phase (psychological stressor) was confined in a restraining cylinder for 2 h, in the second phase (physiological stressor) was forced to swim in a cylindrical container for 20 min, and in the third phase (chemical/endocrinological stressor) was anesthetized with Diethyl ether [9, 17]. After SPS induction, rats were left undisturbed for 2 weeks.

### Drug administration

Fluoxetine hydrochloride (Dr. Abidi Company, Tehran, Iran) in a dose of 10 mg/kg/day was administrated orally for 30 days [18]. The drug was dissolved in drinking water daily. The control groups received water alone. Before drug administration, the volume of water consumption was measured for every cage from the water bottle and converted to a daily dose per weight (mg/kg body). All drug-receiving groups were weighed on the first day of every week.

### Exercise training protocol

Two weeks after SPS, exercise groups were forced to run on a treadmill. At first, habituation to the treadmill apparatus was done by walking on the treadmill at the speed of 3 m/min for 15 min 3 days [19]. After habituation, moderate exercise started. Therefore, rats run on a treadmill with a 0-degree incline, at the speed of 10 m/min for 30 min daily for 5 days per week for 4 weeks. Rats in sedentary groups were placed on the switched-off treadmill for 5 min once a day [20].

### Anxiety-like behavior test

Elevated plus maze (EPM) is a standard and widely used test to assess anxiety-like behavior in rodents [21–23]. It consists of two open (50 × 10) and two enclosed (50 × 10 × 40) arms, crossed in the middle perpendicularly to each other, and a central square (10 × 10 cm). The apparatus is placed at a height of 70 cm above the ground. [21, 23]. Rats were gently placed in the neutral area facing one of the open arms and given 5 min to discover the maze. A total of four paws inside of an arm were used as criteria for entrance. The number of entries into the open arms and the time spent in the open arms were calculated as the indexes of anxiety reduction. Anxiogenic effects selectively decrease the open arm entry and/or open arm time and, in contrast, anxiolytic effects selectively increase the open arm entry and/or open arm time. All sessions were videotaped by a camera.

### Fear extinction test

Fear extinction is a component of cognitive flexibility that is relevant for important psychiatric diseases [24]. The test was done using shuttle box in a sound-protected room. Shuttle-box consists of two dark and light compartments (each 30 × 20 × 20 cm) separated by a guillotine door. The compartments were equipped with the grid floor through which an electric shock (3 s, 1 mA, 50 Hz) was given. Before the test, free ambulation in the shuttle-box for 5 min was allowed in order to be adapted to the context. The extinction test has two phases: the conditioning phase and the extinction phase [25]. The procedure of two phases has been illustrated in our previous study [26]. Briefly, the conditioning phase was carried out in 1 day and then the extinction phases were done over 4 consecutive days (extinctions days 1–4). In the conditioning phase, the animal was placed in the light compartment and the latency for entrance into the dark compartment (the entrance latency) was recorded. After entry into the dark compartment, the animal received a foot shock. Thereafter, the animal was housed in its cage for 24 h, and extinctions were done for 4 days. In extinction phases, the time taken for the animal to enter the dark compartment was recorded as the entrance latency. There is no shock in this phase. The rat was allowed to stay in the dark compartment freely for 180 s, and then it was removed back to the home cage.

### Serum and prefrontal BDNF measurement

On day 49, after the last extinction test, the rats were deeply anesthetized, then decapitated. The trunk blood was centrifuged (4000 g for 20 min) to collect serum. Also, prefrontal cortexes were quickly isolated. Both the serum and prefrontal cortexes were stored at − 80 °C until further analysis [26]. Prefrontal cortex was cut into small pieces and homogenized in phosphate-buffered saline (PBS), pH 7.4 by the homogenizer. The homogenate was centrifuged at 10,000×*g* for 10 min at 4 ℃. Serum and prefrontal cortex BDNF levels were measured by the E-Max ELISA method, according to the manufacturer’s instruction using Rat BDNF ELISA kits (ZellBio GmbH Germany). The absorbance of samples was read at 450 nm by a micro plate reader (RT-2100 C, Germany) and values calculated according to related standard curves. The intra- and inter- assay coefficients of variation were < 10% and < 12%, respectively. The sensitivity of the assay was 1 pg/ml. Total protein concentration was determined by the Bradford method and bovine serum albumin (BSA) was used as a standard [16]. Biochemical parameters were adjusted based on total protein content.

### Statistical analysis

Normality of data was assessed using Kolmogorov–Smirnov test. Data were presented as Mean ± SEM. ANOVA with Tukey HSD, as a post-hoc, was applied to assess the significant difference between the groups. SPSS software version 22 was used for data analysis and p < 0.05 was considered as a significant level.

## Results

### Level of prefrontal and serum BDNF

**Fig. 2:**
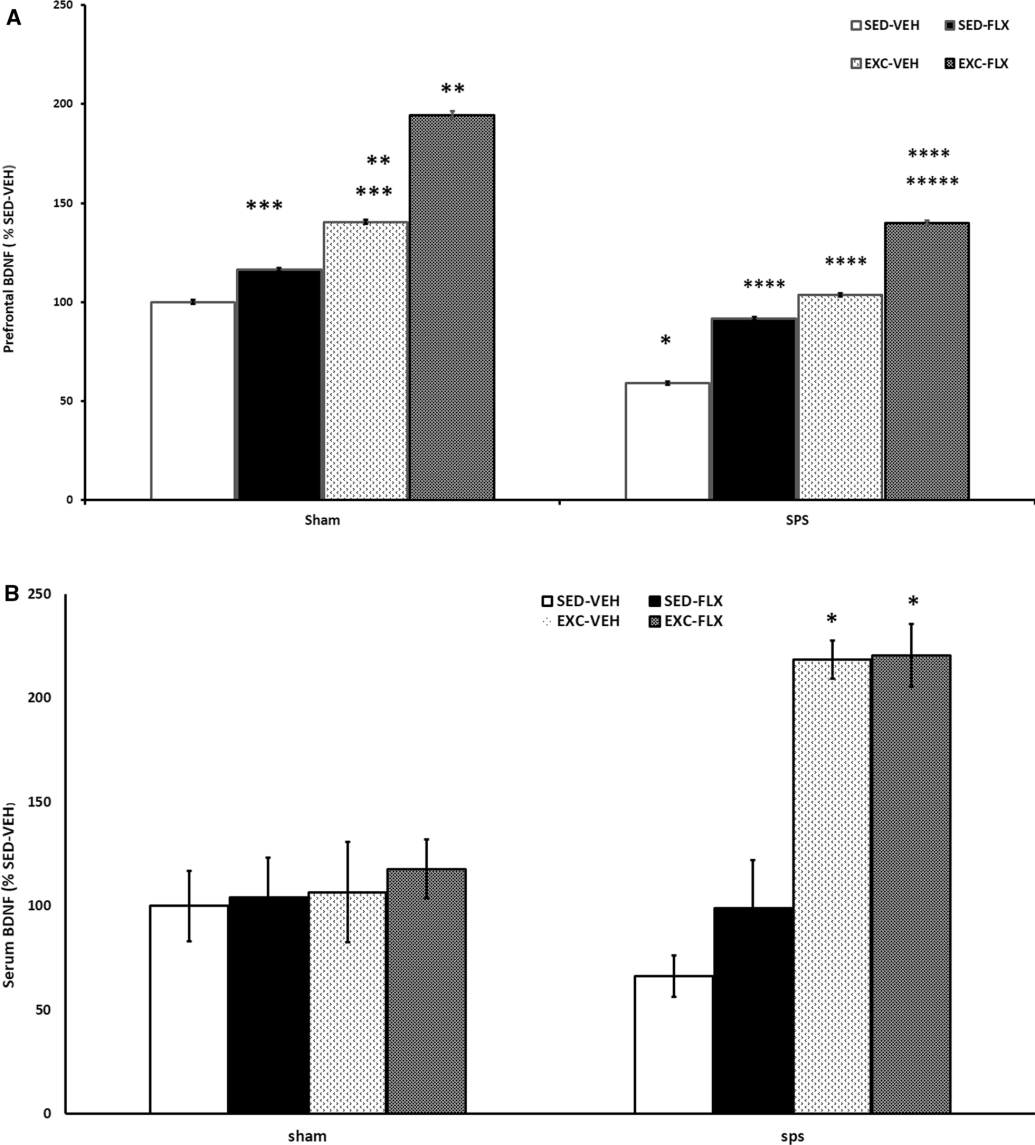
Prefrontal cortex (**A**), and serum BDNF (**B**) in female SPS and sham groups subjected to fluoxetine and treadmill exercise. SPS groups showed reductions in serum and prefrontal cortex BDNF. SPS groups with fluoxetine & treadmill exercise showed increased in serum and Prefrontal cortex BDNF as compared with SPS sedentary groups. **A** *SPS/SED-VEH vs sham/SED-VEH (P = 0.001). **sham/EXE-VEH (P = 0.014) and sham/FLX –EXC (P = 0.0001) vs sham/SED-VEH. ***sham/FLX-SED and sham/EXE-VEH (P = 0.0001) vs sham/FLX –EXC. ****SPS/SED-VEH vs SPS/EXC-FLX (P = 0.0001), SPS/EXC-VEH (P = 0. 001), SPS/SED-FLX (P = 0.003). *****SPS/EXC-FLX vs sham/FLX –EXC (P = 0.0001). **B** *SPS/EXC-VEH (P = 0.015) and SPS/EXC-FLX (P = 0.012) vs SPS/SED-VEH

The findings of the effects of SPS, and treatment on the level of prefrontal BDNF (Fig. 2A), showed a significant treatment effect (F_3,_ 48 = 35.751, P = 0.0001). A significant effect of SPS (F_1,_ 48 = 42.169, P = 0.0001) was observed. The interaction between SPS and FLX was significant (F_3,_ 48 = 4.325, P = 0.009).

The level of prefrontal BDNF was higher in Sham/EXC-FLX in comparison with that in Sham/SED-VEH, Sham /SED-FLX, or Sham/EXC-VEH (P = 0.0001). There was a significant difference between Sham/EXC-VEH and Sham/SED-VEH (P = 0.014), between SPS/SED-VEH and Sham/SED-VEH (P = 0.001), and between SPS/EXC-FLX and Sham/ EXC-FLX (P = 0.0001). Also. SPS/EXC-FLX (P = 0.0001), SPS/EXC-VEH (P = 0. 001), SPS/SED-FLX (P = 0.003) groups had significantly higher prefrontal BDNF in comparison with SPS/SED-VEH group.

The findings of the SPS effects and treatment (Exercise & Fluoxetine) on the level of serum BDNF are illustrated in Fig. 2B. A significant effect of treatment was observed (F_3, 48_ = 4.956, P = 0.004) but not that of SPS (F_1, 48_ = 2.610, P = 0.113). Also, the interaction between SPS and FLX was not significant (F_3, 48_ = 1.778, P = 0.164). There was a significant difference between SPS/EXC-VEH and SPS/SED-VEH (P = 0.015) and between SPS/EXC-FLX and SPS/SED-VEH (P = 0.012).

### Anxiety-related behavior

**Fig. 3:**
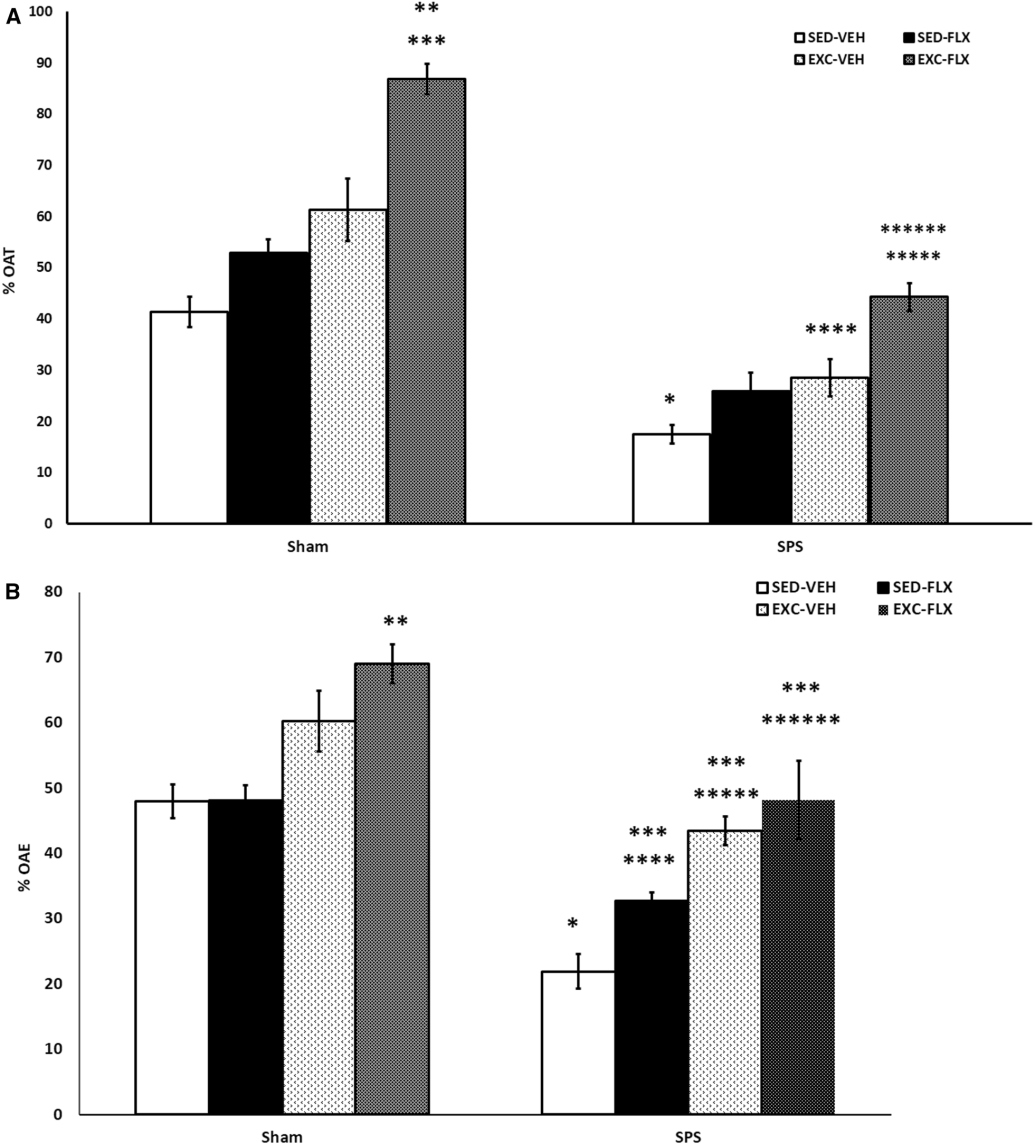
Anxiety-like behaviors in SPS rats subjected to fluoxetine and treadmill exercise as assessed in the elevated plus maze. Exercise and fluoxetine combination groups showed reduction in anxiety level in both groups. SPS groups showed reductions in open arm Time (OAT, A) and open arm Entry (OAE, B). **A** *sham/SED-VEH vs SPS/SED-VEH (P = 0.002). **Sham/EXC-FLX vs sham/SED-VEH (P = 0.0001). ***Sham/EXC-FLX vs Sham/SED-FLX (P = 0.004). ****Sham/EXC-VEH vs SPS/EXC-VEH (P = 0.0001). *****SPS/EXC-FLX vs Sham/EXC-FLX (P = 0.0001). ******SPS/EXC-FLX vs SPS/SED-VEH (P = 0.0001). **B** *SPS/ SED-VEH vs Sham/ SED-VEH (P = 0.0001). **ham/EXC-FLX vs Sham/ SED-VEH (P = 0.046). ***SPS/ EXC-VEH and SPS/ SED-FLX and SPS/ EXC-FLX (P = 0.0001) vs SPS/ SED-VEH. ****SPS/SED-FLX vs Sham/ SED-FLX (P = 0.0001). *****SPS/EXC-VEH vs Sham/ EXC-VEH (P = 0.023). ******SPS/EXC-FLX vs Sham/ EXC-FLX (P = 0.0001)

Figure 3A. shows open arm entry (OAT) percentage. Two way ANOVA on OAT percentage revealed a significant effect for SPS (F_1, 72=_ 116.823, P = 0.0001) and treatment (F_3, 72_ = 32.417, P = 0.0001). However, there was no significant interaction between SPS and treatment (F_3, 72_ 1.318, P = 0.275). According to the results of post-hoc test on the percentage of OAT, there were significant differences between SPS/SED-VEH vs Sham /SED-VEH (P = 0.002), between SPS/EXC-VEH vs Sham/EXC-VEH (P = 0.0001), and between SPS/EXC-FLX vs Sham/EXC-FLX (P = 0.0001). Also, the percentage of OAT in Sham/EXC-FLX group was significantly different from that in Sham/SED-FLX (P = 0.004) and Sham /SED-VEH (P = 0.0001) groups. The difference between SPS/SED-VEH vs SPS/EXC-FLX was significant (P = 0.0001).

Data of open arm entry (OAE) number in the EPM are illustrated in Fig. 3B. A three-way ANOVA on the OAE demonstrated significant main effects of SPS (F_1, 72_ = 74.689, P = 0.0001) and treatment (F_3, 72_ = 10.269, P = 0.0001). There was no significant interaction between SPS and treatment (F_3, 72_ = 1.231, P = 0.305). Between group comparison showed that there was a significant difference in OAE between SPS/ SED-VEH vs Sham/SED-VEH (P = 0.0001), SPS/SED-FLX vs Sham/ SED-FLX (P = 0.0001), SPS/EXC-VEH vs Sham/ EXC-VEH (P = 0.023), and SPS/EXC-FLX vs Sham/EXC-FLX (P = 0.0001) groups. Also, there was significant differences between SPS/SED-VEH vs SPS/EXC-VEH, SPS/SED-VEH vs SPS/SED-FLX, and between SPS/SED-VEH vs SPS/EXC-FLX (P = 0.0001). OAE increased in Sham/EXC-FLX compared to Sham/SED-VEH (P = 0.046) and Sham/SED-FLX (P = 0.040).

### Fear conditioning and extinction

**Fig. 4:**
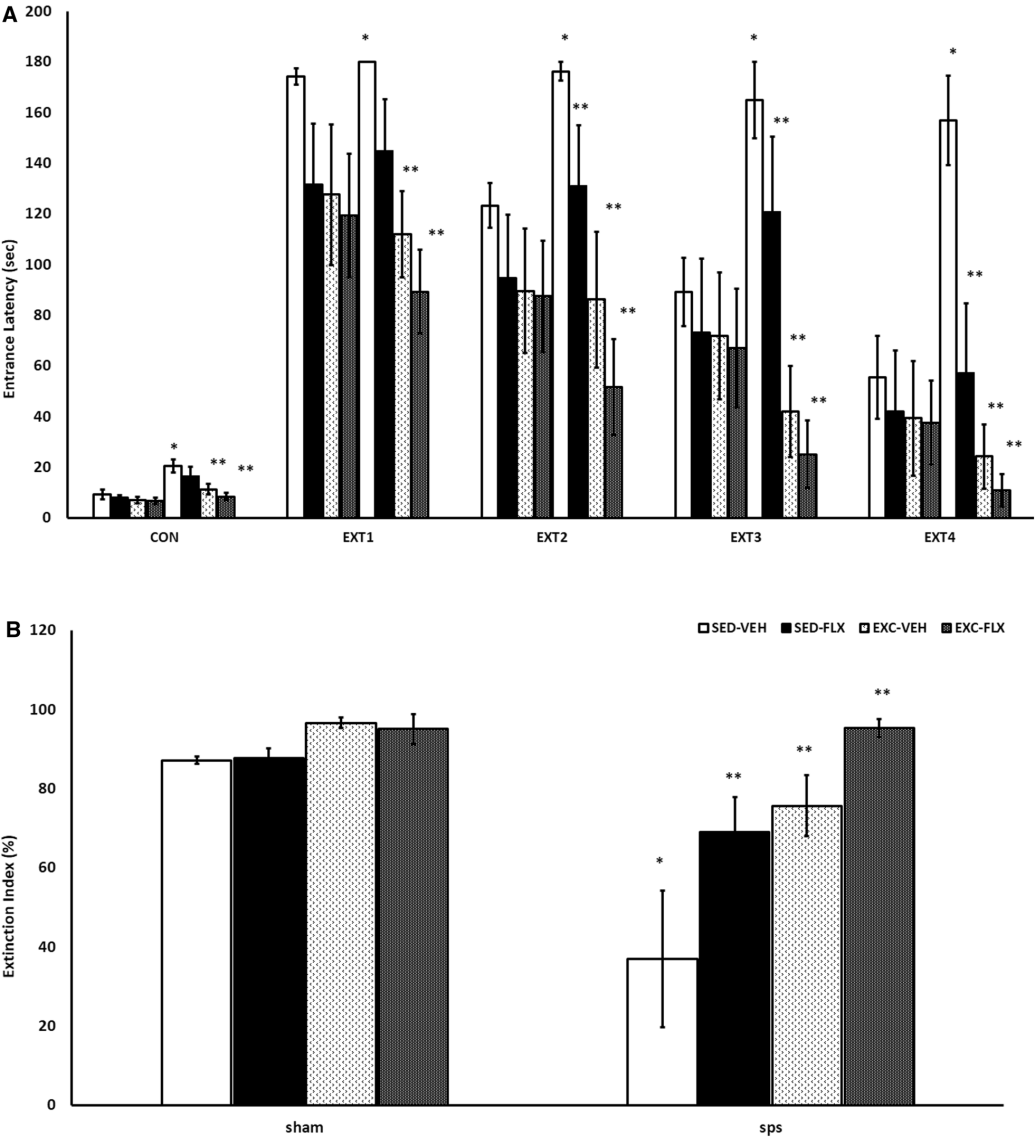
Fear conditioning and extinction index in SPS rats subjected to fluoxetine and treadmill exercise as assessed in extinction test. **A **(Entrance latency time) Exercise and fluoxetine combination groups showed reduction in entrance latency time in both groups. SPS groups showed increased entrance latency time in conditioning phase and in 1th, 2th, 3th, and 4th extinction. In conditioning phase *SPS/SED-VEH vs Sham/SED-VEH (P = 0.001). **SPS/EXC-FLX and SPS/EXC-VEH vs SPS/SED-VEH (P = 0.0001). In 1th extinction **SPS/EXC-FLX, (P = 0.0001) and SPS /EXC-VEH (P = 0.007) vs SPS /SED-VEH. **SPS/SED-FLX vs Sham/SED-FLX (P = 0.002). In 2th extinction*SPS/SED-VEH vs Sham/SED-VEH (P = 0.038). **SPS/EXC-FLX (P = 0.0001), SPS/SED-FLX (P = 0.050), and SPS/EXC-VEH (P = 0.001) vs SPS/SED-VEH. In 3th extinction *SPS/SED-VEH vs Sham/SED-VEH (P = 0.0001). **SPS/EXC-FLX (P = 0.0001), SPS/SED-FLX (P = 0.011) and SPS/EXC-VEH (P = 0.003) vs SPS/SED-VEH. In 4th extinction *SPS/SED-VEH vs Sham/SED-VEH (P = 0.0001). **SPS/EXC-FLX (P = 0.0001), SPS/SED-FLX (P = 0.050) and SPS/EXC-VEH (P = 0.0001) vs SPS/SED-VEH. **B **(Extinction index) *SPS/SED-VEH vs sham/SED-VEH group (P = 0.0001), **SPS/SED-FLX (P = 0.002), SPS/EXC-VEH (P = 0.0001), and SPS/EXC-FLX (P = 0.0001) vs SPS/SED-VEH

Fear conditioning and extinction findings are presented in Fig. 4A. A three –way repeated measurements ANOVA showed significant effects for each of the three factors, treatment (F_3, 48=_ 18.132, P = 0.0001), SPS (F_1, 48=_ 16.345, P = 0.0001) and extinction sessions (F_4, 192=_ 74.689, P = 0.0001) on the entrance latency into the dark box. There were significant interactions among SPS, treatment, and extinction sessions ((F_12, 192=_ 2.038, P = 0.023), between treatment and extinctions sessions (F_12, 192=_5.060, P = 0.0001), and between SPS and extinctions sessions (F_4, 192=_ 3.897, P = 0.005). The interaction between SPS and treatment (F_3, 48=_ 2.259, P = 0.093) was not significant.

In the conditioning phase, SPS/SED-VEH group showed an increase in the entrance latency time compared to Sham/SED-VEH (P = 0.001). Also, the entrance latency was significantly lower in SPS/EXC-FLX (P = 0.0001) and SPS/EXC-VEH (P = 0.0001) in comparison to SPS/SED-VEH. There was a significant difference between SPS/EXC-FLX and SPS/SED-FLX (P = 0.046).

In 1st extinction, the latency time significantly increased in SPS/SED-FLX than in Sham /SED-FLX (P = 0.002). The latency time significantly decreased in SPS/EXC-FLX, (P = 0.0001) and SPS/EXC-VEH (P = 0.007) than in SPS/SED-VEH.

In 2nd extinction, the latency time increased in SPS/SED-VEH in comparison to Sham/SED-VEH (P = 0.038). The significant time reduction was shown in SPS/EXC-FLX (P = 0.0001), SPS/SED-FLX (P = 0.050) and SPS/EXC-VEH (P = 0.001) than in SPS/SED-VEH.

In 3rd extinction, the latency time increased in SPS/SED-VEH in comparison to Sham/SED-VEH (P = 0.0001). The significant time reduction was shown in SPS/EXC-FLX (P = 0.0001), SPS/SED-FLX (P = 0.011) and SPS/EXC-VEH (P = 0.003) than in SPS/SED-VEH.

In 4th extinction, the latency time increased in SPS/SED-VEH in comparison to Sham/SED-VEH (P = 0.0001). The significant time reduction was shown in SPS/EXC-FLX (P = 0.0001), SPS/SED-FLX (P = 0.050) and SPS/EXC-VEH (P = 0.0001) than in SPS/SED-VEH (Fig. 4A).

Extinction index results are shown in Fig. 4B. ANOVA test of extinction index showed a significant effect of treatment (F3, 48 = 11.478, P = 0.0001), SPS (F1, 48 = 10.583, P = 002) and a significant interaction between SPS and treatment (F3_, 48=_ 6.303, P = 0.001).

A comparison between groups showed the extinction percentage in SPS/SED-VEH group was lower than in the sham/SED-VEH group (P = 0.0001). Also extinction percentage in SPS/SED-FLX (P = 0.008), SPS/EXC-VEH (P = 0.001) and SPS/EXC-FLX (P = 0.0001) was higher compared to SPS/SED-VEH.

## Discussion

The results showed that stress exposure led to the reduced OAT and OAE percentage as the markers of anxiety-like behavior. SPS affected entrance latency in fear conditioning and extinction. Also, stress significantly impacted prefrontal BDNF. However, the reduction in serum BDNF following SPS was not statistically significant. Fluoxetine lessened SPS-induced alterations in prefrontal BDNF, fear conditioning, and extinction. However, this drug had no significant effect on OAT and OAE. Exercise alone or in combination with fluoxetine restored the malevolent effects of SPS on anxiety-like behavior, prefrontal and serum BDNF, fear conditioning, and extinction.

The present study confirmed that SPS is able to induce anxiety-like behavior and other PTSD-associated alterations such as BDNF changes and fear conditioning and extinction impairment [27] in female rats. Comparison between present results with our previous findings have shown no sex difference in SPS-induced impairments in behavior, BDNF, fear conditioning, and extinction. Also, therapeutic interventions resulted in somewhat similar effects on both sexes. Some sex differences included greater effect of combination therapy than monotherapies on prefrontal BDNF in males and a greater efficacy of combination therapy than fluoxetine alone on 4th extinction in female rats. Also, unlike male rats, fluoxetine and exercise did not improve OAT in female rats. The findings of our present and past studies [26] have been summarized in the Table 1 to more easily compare male and female rats.

### Effects of exercise, fluoxetine, and a combination of both on prefrontal and serum BDNF in female PTSD rats

The results showed that SPS led to the BDNF reduction in the prefrontal cortex in female rats. The findings were similar to that in male PTSD animals in our previous study [26]. BDNF changes after stress have been frequently reported in several studies [28–31] and our study confirms these findings. Since BDNF has been shown to have an essential role in mediating stress-related pathophysiological changes in the brain [32] these findings are expectable. Impairment in fear extinction is a PTSD marker and BDNF has been reported to be involved in fear memories in the amygdala, hippocampus, and prefrontal cortex [33]. Owing to high sensitivity of the prefrontal cortex to stress and its modulation role in many behavioral and physiological responses to stress [34], BDNF alterations in the prefrontal cortex after SPS is an important finding.

Comparison between male and female rats showed no sex difference in BDNF reduction after SPS. Although some studies such as that of Aykaç revealed that BDNF alteration after SPS has sex differences [28, 30], our studies showed similar results in prefrontal BDNF between male and female rats. This finding was partly in agreement with the results of a systematic review and meta-analysis study that showed no significant effect of sex on peripheral BDFN in PTSD patients [35].

In Aykaç and colleagues' study, no significant difference was found between males and females in cortical BDNF levels after the social isolation test, however, female rats had lower amygdala BDNF than males after the test [28]. Based on the mentioned study, it is probable that the difference in findings between males and females originated from the region of the brain collected for BDNF measurement. In other words, stress-induced BDNF alteration in the distinct brain parts differs in a sex-dependent manner [28]. Since the prefrontal region has been shown to be one of the involved parts in PTSD, the present study assessed prefrontal BDNF and similar results were found in males and females.

Fluoxetine and exercise individually or in combination were able to restore prefrontal BDNF in female animals. These findings were similar to those in male animals [26]. However, there were some sex differences between males and females in the therapeutic potency of fluoxetine alone, exercise alone, and the combination of both. In female PTSD rats, there was no difference between the effect of monotherapies and combination therapy on prefrontal BDNF while in male PTSD rats, combination therapy was more potent than monotherapies.

It has been shown that both the antidepressant drug fluoxetine and exercise are able to increase BDNF expression in the brain exposed to stress [26, 28, 36], and our results are consistent with these findings. In this regard, exercise induces BDNF enhancement during extinction consolidation and, consequently, reduces threat expectancies following reinstatement in PTSD [37]. Also, it is asserted that the likely role of drugs such as antidepressants on fear extinction improvement is modulated through the BDNF-TrkB system [33]. Taken together, in studies designed for PTSD treatment with antidepressants and exercise, BDNF modulation is considered as one of the involved mechanisms.

Although sex differences in the therapeutic effects of fluoxetine or exercise on BDNF have been reported in some studies [28, 38, 39], in our studies, fluoxetine or exercise led to similar therapeutic outcomes in both sexes. The only sex difference in prefrontal BDNF was that the combination of fluoxetine and exercise was more potent than monotherapies in male but not in female rats. Interestingly, the combination of fluoxetine and exercise was able to enhance prefrontal BDNF to a higher level than the normal range in male SPS rats (data was not shown). In Aykaç and colleagues' study, the fluoxetine effects on BDNF were different between the two groups depending on the assessed region of the brain. For example, fluoxetine was able to restore BDNF in the female’s hippocampus but not in the male’s. Similar results have been reported by Mitic and colleagues [38]. Accordingly, it has been asserted that fluoxetine effects on stressed subjects are also in a brain region-specific manner [40]. Therefore, it is likely that inconsistency in the results of BDNF in male and female subjects is due to differences in the assessed regions of the brain.

In the present study, unlike the prefrontal BDNF, serum BDNF did not show a statistically significant difference between SPS and non-SPS rats. Due to difficulty in crossing the blood–brain barrier [41], peripheral BDNF may not be an accurate biomarker to reflect slight alterations in brain BDNF [42]. However, according to the results of a meta-analysis study [35], it was probable that measuring plasma BDNF instead of serum BDNF would produce more conclusive results.

### Effects of exercise, fluoxetine, and a combination of both on fear extinction in female PTSD rats

Deficient fear extinction is considered as one of the hallmarks of PTSD symptoms and the present study showed that SPS impaired the extinction of conditioned fear in female rats. Entrance latency decreased in all intervention groups, including fluoxetine alone, exercise alone, and fluoxetine plus exercise over time. These results suggest that both monotherapy and combination therapy were able to facilitate fear memory extinction in female SPS rats. Comparison between male and female SPS rats indicated somewhat similar results in terms of the effectiveness of the interventions. However, it is mentionable that in female SPS rats, unlike in males, combination therapy showed greater efficacy in reducing entrance latency on the 4th day than fluoxetine alone.

Although some studies showed that stress-exposed female rats have more disrupted performance in the fear extinction learning [5, 29], our study did not reveal a distinct difference between males and females in the impact of interventions, except greater efficacy of combination therapy than fluoxetine alone in female rats.

The present study showed that the interventions were able to enhance BDNF in the prefrontal regions in both male and female SPS rats. Besides, they improved fear extinction over time in both sexes. These findings support the documents reporting that the positive effect of interventions such as exercise on fear extinction learning is mediated by increasing BDNF [37, 43, 44]. Since BDNF plays a role in the fear extinction enhancement, targeting the impaired extinction in anxiety disorders such as PTSD via BDNF signaling may be an important and novel way to enhance treatment efficacy [33].

There was a slight sex difference in terms of the therapeutic effect of fluoxetine on the 4th extinction, but not the extinction index. The results in female SPS rats showed that the effect of fluoxetine was satisfying but it was less than that of combination therapy. Previous studies also reported that fluoxetine resulted in a different effect between male and female groups [9, 10].

### Effects of exercise, fluoxetine, and a combination of both on anxiety-like behaviors in female PTSD rats

PTSD is an anxiety disorder and SPS is known as an valid rodent model of it [45]. The results of EPM tests showed that SPS was able to induce anxiety-like behavior in female rats. SPS-exposed rats showed fewer entries into the open arms and lower percentage of open arm time in comparison to non-SPS rats.

Fluoxetine alone could not show a significant therapeutic effect on anxiety-like behaviors assessed by EPM in female rats. However, exercise was able to improve the percentage of open arm entries but had no significant effect on the percentage of open arm time in female SPS rats. Combination therapy was more potent than monotherapies and improved both parameters including OAT and OAE in the female rats as it did in male rats. Comparison of the present results with our findings of the previous study showed that fluoxetine alone and especially exercise alone led to better effects in male rats than that in females. These results can be supported to some extent by Whitworth and colleagues’ study that showed active men have significantly lower PTSD symptoms than active women [6]. Morgan and colleagues’ study showed exercise-induced anxiety behaviors in young female mice [15]. Regarding the role of gonadal hormones on the stress-related pathways such as the prefrontal cortex–amygdala pathway [46], it was not surprising to see some different findings between males and females. Although the present study has shown many similarities between male and female rats regarding the effects of treatments, some inconsistent findings support the opinion of the role of sex and hormones on stress management and the need for individualized treatments considering sex effects [7, 15, 47, 48].

Fluoxetine alone was not able to induce statistically significant improvement in anxiety behavior in female rats, however, it resulted in a subtle increase in OAE and OAT. Therefore, it may show a synergic effect if co-administrated with other treatments, as this synergic effect was revealed in combining fluoxetine with exercise. We can claim the same for exercise alone that slightly increased OAE. Potentiation of the therapeutic effect of fluoxetine in combination with exercise was also reported in Gobinath and colleagues' study on postpartum depression in female rats. Their study showed that fluoxetine augmented neurogenesis only when combined with exercise [13]. Taken together, in some parameters, the greater therapeutic effects were shown in male SPS rats than in female rats. These findings can partly assert sex-specific psychopharmacology in psychological disorders. While there were some sex differences in response to fluoxetine alone or exercise alone, combination therapy was able to restore all parameters affected by PTSD in both sexes.

It is worth mentioning that although some sex differences in the effect of interventions on stress-exposed subjects [6, 28] have been reported, the accurate conclusion needs to compare intervention-induced changes with the normal scores in males and females separately. It is probable that the baseline score is different in a sex-specific manner and consequently leads to different final results between males and females. For example, in our study OAT and OAE percentage in sham groups, as the baseline scores, were different between males and females (data was not shown). Regarding these different findings in the present study, the changes after SPS induction and following intervention have been compared with the base scores in each sex separately.

## Conclusion

In female rats, there was no significant difference between the effects of combination therapy and exercise alone in SPS-induced deficits except the increased OAT. In male rats, exercise alone showed more potency and the improved OAT. Therefore, exercise as a safe and cost-effective intervention can be considered as a complementary efficient option for PTSD treatment in both sexes. Although the majority of findings related to the efficacy of treatments were comparable between males and females, there were some sex differences. Therefore, to achieve better outcomes, considering the differences between genders is recommended in the treatment of PTSD patients.

## Limitations

In the present study, we did not determine the phase of estrous cycles in female animals. Since the level of sex hormones can influence behavioral and physiological events, the results may be impacted by the hormonal changes of the menstrual cycle. Measurement of BDNF was limited to the prefrontal cortex, which, although is an important structure involved in PTSD, is not the only one. Therefore it would have been better to assess other brain structures involved, such as the hippocampus, for more convincing results in female PTSD. Moreover, we did not analyze the correlation between behavioral and molecular data due to a large number of tests. These limitations should be considered in future studies.

**Table 1:** Effects of fluoxetine, exercise, and the combination of both, in male and females rats, comparison present results with reported data in previous study [26]

Assessed parameter	Interventions	P value	
		Male	Female
Prefrontal BDNF	Fluoxetine alone (SPS/SED-VEH vs SPS/SED-FLX)	0.019	0.003
	Exercise alone (SPS/SED-VEH vs SPS/EXC-VEH)	0.000	0.001
	Combination therapy (SPS/SED-VEH vs SPS/EXE-FLX)	0.000	0.000
	Combination therapy VS fluoxetine alone (SPS/SED-FLX vs SPS/EXE-FLX)	0.001	0.565
	Combination therapy VS exercise alone (SPS/EXC-VEH vs SPS/EXE-FLX)	0.025	0.761
4th extinction	Fluoxetine alone (SPS/SED-VEH vs SPS/SED-FLX)	0.000	0.05
	Exercise alone (SPS/SED-VEH vs SPS/EXC-VEH)	0.000	0.000
	Combination therapy (SPS/SED-VEH vs SPS/EXE-FLX)	0.000	0.000
	Combination therapy VS fluoxetine alone (SPS/SED-FLX vs SPS/EXE-FLX)	0.26	0.008
	Combination therapy VS exercise alone (SPS/EXC-VEH vs SPS/EXE-FLX)	0.56	0.51
Extinction index	Fluoxetine alone (SPS/SED-VEH vs SPS/SED-FLX)	0.000	0.008
	Exercise alone (SPS/SED-VEH vs SPS/EXC-VEH)	0.000	0.001
	Combination therapy (SPS/SED-VEH vs SPS/EXE-FLX)	0.000	0.000
	Combination therapy VS fluoxetine alone (SPS/SED-FLX vs SPS/EXE-FLX)	0.34	0.054
	combination therapy VS exercise alone (SPS/EXC-VEH vs SPS/EXE-FLX)	0.75	0.13
OAT%	Fluoxetine alone (SPS/SED-VEH vs SPS/SED-FLX)	0.000	0.823
	Exercise alone (SPS/SED-VEH vs SPS/EXC-VEH)	0.004	0.528
	Combination therapy (SPS/SED-VEH vs SPS/EXE-FLX)	0.001	0.000
	Combination therapy VS fluoxetine alone (SPS/SED-FLX vs SPS/EXE-FLX)	1	− 0.004
	Combination therapy VS exercise alone (SPS/EXC-VEH vs SPS/EXE-FLX)	1	− 0.019
OAE	Fluoxetine alone (SPS/SED-VEH vs SPS/SED-FLX)	0.11	0.063
	Exercise alone (SPS/SED-VEH vs SPS/EXC-VEH)	0.001	0.0001
	Combination therapy (SPS/SED-VEH vs SPS/EXE-FLX)	0.000	0.000
	Combination therapy VS fluoxetine alone (SPS/SED-FLX vs SPS/EXE-FLX)	0.000	0.322
	Combination therapy VS exercise alone (SPS/EXC-VEH vs SPS/EXE-FLX)	0.83	0.965

## Data Availability

The datasets used and/or analyzed during the current study are available from the corresponding author on reasonable request.
